# A workflow for assessing antibody-drug conjugate target expression on circulating tumour cells from triple-negative breast cancer and epithelial ovarian cancer patients

**DOI:** 10.1016/j.jlb.2026.100479

**Published:** 2026-06-30

**Authors:** Brian D. Henderson, Lauren McMahon, Faye Lewis, Caroline Marion, Sinead Hurley, Kathy Gately, Irene Narinda, Marika Kanjuga, Lucy Norris, Cara Martin, Neil Conlon, Lorraine O’Driscoll, Feras Abu Saadeh, Catherine O’Gorman, Patrick J. Maguire, Waseem Kamran, Elzahra Ibrahim, Karen A. Cadoo, Niamh Coleman, John J. O’Leary, Mark P. Ward, Sharon A. O’Toole

**Affiliations:** a Department of Histopathology, School of Medicine, Trinity College Dublin, Dublin, Ireland; b Department of Obstetrics and Gynaecology, School of Medicine, Trinity College Dublin, Dublin, Ireland; c Trinity St. James's Cancer Institute, St James’s Hospital, Dublin, Ireland; d Department of Clinical Medicine, Trinity Translational Medicine Institute, School of Medicine, Trinity College Dublin, Ireland; e National Institute for Cellular Biotechnology, School of Biotechnology, Dublin City University, Dublin, Ireland; f School of Pharmacy and Pharmaceutical Sciences, Trinity College Dublin, Ireland; g Trinity Biomedical Sciences Institute, Trinity College Dublin, Ireland; h Division of Gynaecological Oncology, St James’s Hospital, Dublin, Ireland; i The Haematology, Oncology and Palliative Care (HOPe) Directorate, St. James’s Hospital, Dublin, Ireland; j Department of Pharmacology and Therapeutics, School of Medicine, Trinity College Dublin, Ireland

**Keywords:** Antibody-drug conjugates, Triple-negative breast cancer, Epithelial ovarian cancer, Circulating tumour cells, Epitope-independent enrichment, Multiplex staining

## Abstract

**Background:**

Antibody–drug conjugates (ADCs) are transforming the therapeutic landscape of solid tumours. Both patient selection and ADC efficacy in triple-negative breast cancer (TNBC) and epithelial ovarian cancer (EOC) are impacted by target antigen expression, which is often heterogeneous. Single tissue biopsies cannot capture this spatial heterogeneity, creating a need for real-time assessment to guide treatment. Circulating tumour cells (CTCs) may serve as minimally invasive biomarkers that can reflect ADC target expression. The aim of this study was to assess the detectability of clinically relevant ADC targets on CTCs in TNBC and EOC.

**Methods:**

ADC targets TROP-2 (TNBC) and FRα (EOC) were selected, along with emerging targets PD-L1 and CLDN6. Flow cytometry was used to characterise surface target antigen expression in three TNBC (MDA-MB-231, MDA-MB-468, HCC1937) and three EOC (SKOV3, OVCAR3, Kuramochi) cell lines. Cell spike-in assays using the Parsortix® system were performed to optimise in-cassette fixation and staining conditions, which were then applied to patient-derived samples for CTC enrichment and immunostaining.

**Results:**

Flow cytometry revealed heterogeneous antigen expression across cell lines. In TNBC, TROP-2 was uniformly expressed including in EpCAM-low MDA-MB-231 cells, whereas FRα was minimal or absent. PD-L1 was highly expressed in MDA-MB-231 and HCC1937 but low in MDA-MB-468. In EOC, FRα and TROP-2 were detectable in SKOV3 and OVCAR3 but low or absent in Kuramochi cells. Spike-in assays confirmed in-cassette detection of TROP-2, FRα, and PD-L1 following Parsortix® enrichment. In patient samples, TROP-2+ CTCs were identified in TNBC and FRα+ CTCs in EOC.

**Conclusion:**

This study demonstrates the feasibility of real-time ADC target assessment on CTCs using epitope-independent enrichment. TROP-2 shows additional utility for detecting CTCs with low EpCAM expression in TNBC. Incorporating ADC target markers into CTC workflows may enable monitoring of target expression, with potential to guide therapeutic decision-making in TNBC and EOC.

## Graphical abstract

**Figure:**
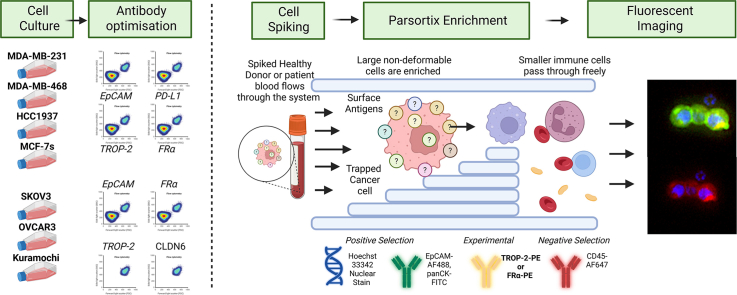

## 1 Introduction

Triple-negative breast cancer (TNBC) and epithelial ovarian cancer (EOC) represent two of the most aggressive and therapeutically challenging solid tumours, characterised by high rates of recurrence, chemoresistance, and poor overall survival [1,2]. Antibody-drug conjugates (ADCs) have emerged as a transformative class of targeted therapies that combine a monoclonal antibody specific for a tumour-associated antigen (TAA) with a potent cytotoxic payload via a chemical linker [3,4]. A growing number of ADCs have been approved by the U.S. Food and Drug Administration (FDA) and the European Medicines Agency (EMA), spanning multiple TAAs, including human epidermal growth factor receptor 2 (HER2), tissue factor (TF), trophoblast cell-surface antigen 2 (TROP-2), hepatocyte growth factor receptor (MET), folate receptor alpha (FRα), and nectin-4 (NECTIN4) [5]. In EOC, mirvetuximab soravtansine (MIRV), a FRα-directed ADC, demonstrated superior efficacy over chemotherapy in FRα-high platinum-resistant disease in the phase III MIRASOL trial [6]. In metastatic TNBC, sacituzumab govitecan (SG), a TROP-2-targeted ADC, almost doubled progression-free and overall survival compared to chemotherapy in the phase III ASCENT trial [7,8].

Despite these advances, a major challenge is that adequate target antigen expression in the tumour remains one of the key determinants of ADC efficacy [3]. Antigen expression in TNBC and EOC is often heterogeneous and can evolve under therapeutic pressure. In breast cancer, TROP-2 shows inter- and intra-patient variability, with mechanistic and clinical sequencing data indicating that SG resistance can arise due to mutations that reduce membrane localisation and reduce antibody epitope affinity [9]. Reductions in membrane HER2 have likewise been linked to diminished trastuzumab emtansine (T-DM1) activity [10]. Patients are typically selected for ADC therapy based on a single tissue biopsy, however, this invasive snapshot may not capture spatial heterogeneity in antigen expression, which may be amplified by selective evolutionary pressures from prior treatments [11,12]. Moreover, significant variability in optimal expression cut-offs has been observed across clinical trials, even for the same antigen [13]. Patient selection for MIRV, for example, requires ≥75% of viable tumour cells staining with ≥2+ intensity by the VENTANA FOLR1 RxDx IHC assay, a threshold met in approximately 32–36% of high grade serous ovarian cancer (HGSOC) patients [14,15]. While higher FRα expression generally predicts better response to FRα-targeting ADCs, clinical benefit is also observed in patients with intermediate expression, as is observed with the HER2-targeting ADC trastuzumab deruxtecan [16,17]. This underscores the need for more comprehensive approaches to biomarker assessment and patient stratification. Given the ubiquitous expression of TROP-2 in many epithelial tumours (approximately 90% of TNBC), no formal cut-off for TROP-2 positivity is currently used to select patients for SG, indicating the need for better predictive biomarkers, particularly as ADCs are incorporated into first-line treatment strategies [18]. Moreover, emerging evidence suggests that combining ADCs with immune checkpoint inhibitors may improve outcomes in biomarker-selected subsets, highlighting the potential value of co-assessing targets such as PD-L1 alongside ADC antigens for combination therapy stratification [19].

Circulating tumour cells (CTCs) are tumour-derived cells shed from primary and metastatic sites into the peripheral blood, and their enumeration has been established as a prognostic biomarker across solid tumours [20,21]. In metastatic breast cancer (mBC), CTC detection correlates with disease progression and survival, and a similar relationship has been described in EOC, where CTCs are associated with disease burden and treatment response [21,22]. Our group has recently identified CTCs as a prognostic indicator of progression-free survival in advanced HGSOC [23]. Beyond enumeration, CTC-based molecular profiling offers additional opportunities to characterise therapeutically relevant phenotypes, capture tumour heterogeneity and identify actionable therapeutic targets. By providing a minimally invasive, real-time ‘liquid biopsy’, this approach enables dynamic assessment of tumour biology and complements the static snapshot afforded by tissue biopsy, supporting precision oncology in solid tumours [24].

However, a key consideration for CTC-based biomarker assessment is the method of enrichment. EpCAM-based capture platforms, such as CellSearch®, are widely used but may fail to detect CTCs that have undergone epithelial-to-mesenchymal transition (EMT), a process by which cancer cells downregulate epithelial markers including EpCAM to acquire a more invasive and migratory phenotype [25]. This limitation is particularly relevant for CTCs from TNBC and EOC, where EMT is frequently observed. Label-independent enrichment technologies that isolate CTCs based on biophysical properties, such as size and deformability, offer an antigen-agnostic alternative that can capture phenotypically heterogeneous CTC populations regardless of surface marker expression. The Parsortix® microfluidic system (CelLBxHealth plc) is one such platform and has been previously applied to quantify HER2 expression on CTCs from mBC patients using an EpCAM-independent approach [26].

The use of CTC molecular profiles to guide targeted therapy is growing. The DETECT III trial demonstrated improved overall survival in patients with tumour HER2-mBC but HER2+ CTCs who were treated with lapatinib [27]. Similarly, FRα+ CTCs have been detected in patients with ovarian, breast, and other epithelial cancers, suggesting their potential utility as a biomarker for diagnosis and treatment monitoring [[28], [29], [30]]. TROP-2 is broadly expressed in breast cancer, and recent work has confirmed its detection on CTCs from patients with both early and metastatic TNBC [31]. These studies collectively support the rationale for extending CTC-based molecular profiling to ADC target assessment, enabling real-time monitoring of target availability that may inform patient selection, treatment, and the identification of resistance mechanisms.

The ASCENT-04/KEYNOTE-D19 trial demonstrated that SG combined with pembrolizumab improved outcomes compared to chemotherapy plus pembrolizumab in previously untreated PD-L1+ advanced TNBC [19], and immunotherapy is now being incorporated into first-line neoadjuvant regimens for TNBC [32]. The ability to co-assess ADC targets alongside immune markers such as PD-L1 on the same CTC population could further support patient stratification for combination regimens. Additionally, novel ADC targets such as claudin-6 (CLDN6), an oncofetal tight-junction protein, are being pursued by next-generation ADCs (TORL-1-23; DS-9606a) and cellular therapies (BNT211-01), with early clinical response across CLDN6+ solid tumours [33,34].

This study aims to develop a workflow to evaluate the expression of TROP-2, FRα, PD-L1 and claudin-6 on CTCs in TNBC and EOC using the Parsortix® epitope-independent enrichment system and multiplex immunofluorescence.

## 2 Materials and methods

### 2.1 Cell culture and spiking experiments

Cell lines: TNBC lines (HCC1937, MDA-MB-468, MDA-MB-231) were used and a Luminal A breast cancer line (MCF-7) (EpCAM-high), which served as a control for CTC device optimisation. HGSOC lines (OVCAR3, Kuramochi), and an additional EOC line (SKOV3) were used. Cells were cultured in their respective base media: DMEM (MCF-7, MDA-MB-468), RPMI (HCC1937, MDA-MB-231, OVCAR3, Kuramochi), and McCoy's 5A (SKOV3) (Gibco, UK). All media were supplemented with 10% foetal bovine serum and 1% penicillin-streptomycin (Sigma-Aldrich, Dublin, Ireland). Cultures were maintained at 37°C in a humidified incubator with 5% CO_2_. 2000 cells were spiked into 7.5 mL of healthy donor whole blood for immunofluorescent optimisation.

### 2.2 Flow cytometry analysis of ADC targets

Cells were collected at a density of 0.2 x 10^6^ cells/mL in flow cytometry tubes. Cells were stained with zombie-aqua viability dye (423101) and surface markers epithelial cell adhesion molecule (EpCAM) (Alexa Fluor 488; BioLegend USA, #32410, 1:100), FRα (PE; BioLegend USA, #908304, 1:100), TROP-2 (PE; BioLegend USA, #363804, 1:100), PD-L1 (PE; BioLegend, USA, #393608, 1:100) (Biolegend, USA) and claudin-6 (PE; Santa Cruz Biotechnology, USA, sc-393671, 1:100). Events were acquired using the CANTO II (BD Biosciences, UK) flow cytometer and the Cytek® Northern Lights system. Data were analysed using FlowJo software (BD Biosciences, UK).

### 2.3 Parsortix® CTC enrichment and detection

The Parsortix® PR1 microfluidic system (CelLBxHealth plc, UK) was used for CTC enrichment. Samples were processed within 4 h of collection. 2000 cells were spiked into each sample. Following enrichment, captured cells were fixed with 4% paraformaldehyde (Sigma-Aldrich, Ireland) and in-cassette staining was performed. TNBC cells were stained with an antibody cocktail for positive selection: anti-EpCAM (9C4, Alexa Fluor 488; BioLegend, USA; #324210), anti-pan-cytokeratin (panCK) (CK3-6H5, FITC, Miltenyi Biotec, Germany; #ab192643), and anti-epidermal growth factor receptor (EGFR) (AY13, Alexa Fluor 488, BioLegend, USA, #352908). Leukocytes were excluded using anti-CD45 (HI30, Alexa Fluor 647, BioLegend, USA, #304018). Nuclear staining was performed with 20 mM Hoechst 33342 (Thermo Scientific, USA, #815-968-0747). Cells were stained with experimental antibodies against TROP-2 (NY18, PE; BioLegend, USA; #363804) or PD-L1 (MIH2, PE; BioLegend, USA, #393608). All antibodies were diluted 1:120 in Inside Perm (Inside Stain Kit, MACS Miltenyi Biotec, Germany; #130-090-477).

Ovarian cells were stained with an antibody cocktail for positive selection: anti-EpCAM (9C4, Alexa Fluor 488; BioLegend, USA; #324210) and anti-panCK (CK3-6H5, FITC, Miltenyi Biotec, Germany). Nuclear staining was performed with 20 mM Hoechst (Thermo Scientific, USA; #815-968-0747). Cells were stained with experimental antibodies against FRα (LK26, PE; BioLegend, USA; #908304) and TROP-2 (NY18, PE; BioLegend, USA; #363804). Leukocytes were excluded using anti-CD45 (HI30, Alexa Fluor 647, BioLegend, USA; #304018). All antibodies were diluted 1:120 in Inside Perm (Inside Stain Kit, MACS Miltenyi Biotec, Germany; #130-090-477).

### 2.4 Visualisation and analysis by immunofluorescence microscopy

Parsortix® cassettes were viewed using an EVOS® FL digital inverted fluorescence microscope (Invitrogen™, USA). For EOC, CTC positivity was defined as the presence of ≥1 EpCAM+/panCK+/Hoechst + cell that was CD45−and exhibited round, intact morphology with a large nucleus-to-cytoplasm ratio. EGFR was included within the tumour-identification (pan-CK/EpCAM) channel for TNBC to increase detection sensitivity, accounting for the reduced and heterogenous EpCAM expression characteristic of basal-like and EMT-associated TNBC phenotypes.

### 2.5 Patient cohort

Peripheral blood samples were collected from patients with metastatic TNBC (n = 6) and advanced (stage III) EOC (n = 3) at St. James’s Hospital, Dublin. All patients included in this study gave full and informed written consent (St. James’s Hospital/Tallaght University Hospital Joint Research Ethics Committee (ID:2095)). Blood (7.5 mL) was collected in Vacutainer® K2E K2EDTA tubes (Greiner Bio-One, Austria) and processed within 4 h of collection.

### 2.6 Statistical analysis

Flow cytometry data are presented as mean ± standard error of the mean (SEM) from three independent experiments. Statistical analyses were performed using one-way ANOVA followed by Tukey’s post hoc multiple comparisons test in Prism v10.0 (GraphPad Prism, San Diego, California, USA). Significance was considered to be p < 0.05.

## 3 Results

### 3.1 Expression of ADC targets in TNBC cell lines

**Fig. 1:**
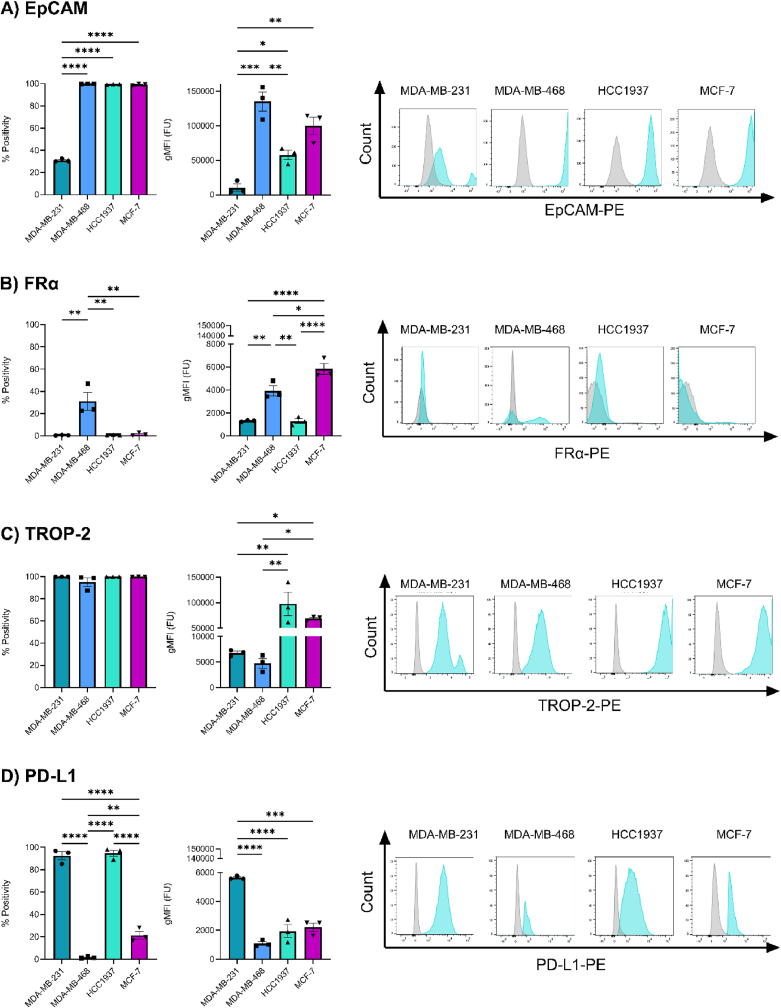
**TROP-2 is highly expressed across TNBC cell lines, whereas high expression of PD-L1 is only observed in MDA-MB-231 and HCC1937 cell lines**. Bar charts showing % frequencies (**left**), or geometric mean fluorescence intensity unit (gMFI (FU), (**middle**) of TNBC cell lines (MDA-MB-231, MDA-MB-468 and HCC1937) and the Luminal A control cell line (MCF-7) expressing **(A)** EpCAM, **(B)** FRα, **(C)** TROP-2 and **(D)** PD-L1. Representative histograms with fluorescence minus one (FMO) (grey) are overlaid with antibody-stained samples (blue) (**right**). One-way ANOVA was used followed by a Tukey’s post hoc test and presented as mean ± SEM from three independent experiments, ∗p < 0.05, ∗∗p < 0.01, ∗∗∗p < 0.001, ∗∗∗∗p < 0.0001.

Flow cytometry analysis of TNBC cell lines (MDA-MB-231, MDA-MB-468, HCC1937) and the Luminal A breast control cell line MCF-7 revealed marked heterogeneity in expression of ADC targets. EpCAM was highly expressed on MDA-MB-468, HCC1937 and MCF-7 cells, each demonstrating >95% positivity and high geometric mean fluorescence intensity (gMFI) [Fig. 1A]. In contrast, EpCAM positivity was significantly lower in MDA-MB-231 cells (31%) compared to all other cell lines (p < 0.0001 for all comparisons; n = 3), with correspondingly reduced gMFI (10213 FU) [Fig. 1A]. FRα expression was largely absent across all TNBC cell lines. MDA-MB-468 was the only TNBC line with appreciable positivity (31%), which was significantly higher than MDA-MB-231 (0.8%, p = 0.0027), HCC1937 (0.8%, p = 0.0029) and MCF-7 (2.9%, p = 0.0034; n = 3) [Fig. 1B]. TROP-2 was universally expressed across all four cell lines, with >95% positivity in each (n = 3) [Fig. 1C]. Despite similar positivity rates, expression intensity varied considerably. HCC1937 cells displayed the highest gMFI (97843 FU), which was significantly greater than MDA-MB-231 (6789 FU, p = 0.0023) and MDA-MB-468 (4770 FU, p = 0.0020). Similarly, MCF-7 gMFI (69080 FU) was significantly higher than MDA-MB-231 (p = 0.0209) and MDA-MB-468 (p = 0.0177; n = 3) [Fig. 1C]. PD-L1 expression was heterogeneous across the cell lines. MDA-MB-231 and HCC1937 cells demonstrated the highest positivity (92.4% and 94.7%, respectively), both significantly greater than MDA-MB-468 (1.6%) and MCF-7 (21.4%) (p < 0.0001 for all comparisons; n = 3) [Fig. 1D]. MDA-MB-231 cells also exhibited the highest PD-L1 gMFI (5638 FU), which was significantly higher than MDA-MB-468 (1096 FU, p < 0.0001), HCC1937 (1938 FU, p < 0.0001) and MCF-7 (2215 FU, p = 0.001; n = 3) [Fig. 1D].

### 3.2 Expression of ADC targets in EOC cell lines

**Fig. 2:**
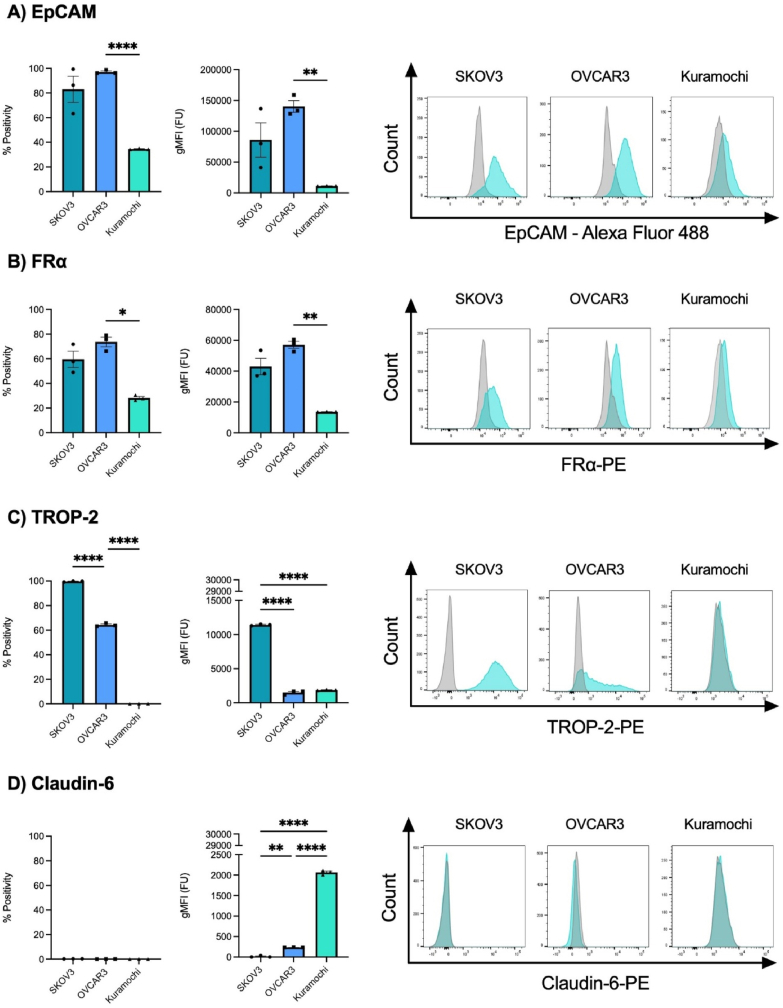
**Differential expression of FRα and TROP-2 across EOC cell lines.** Bar charts showing % frequencies (**left**), or geometric mean fluorescence intensity unit (gMFI (FU), **middle**) of SKOV3, OVCAR3 and Kuramochi ovarian cancer cell lines expressing **(A)** EpCAM, **(B)** FRα, **(C)** TROP-2 and **(D)** Claudin-6. Representative histograms with FMO (grey) are overlaid with antibody-stained samples (blue) (**right**). One-way ANOVA was used followed by a Tukey’s post hoc test and presented as mean ± SEM from three independent experiments, ∗p < 0.05, ∗∗p < 0.01, ∗∗∗∗p < 0.0001.

In EOC cell lines (SKOV3, OVCAR3, Kuramochi), distinct target expression profiles were observed. EpCAM was highly expressed in OVCAR3 (97%) and SKOV3 (83%) cells. Kuramochi cells displayed significantly lower expression compared to OVCAR3 (34.5%); p < 0.0001; n = 3) [Fig. 2A]. gMFI followed the same trend, with OVCAR3 (140246 FU) significantly exceeding Kuramochi (11127 FU) (p = 0.01, n = 3). SKOV3 displayed intermediate expression (85843 FU) [Fig. 2A]. FRα also showed variable expression among the EOC cell lines. OVCAR3 and SKOV3 both demonstrated moderate to high positivity (74% and 60%, respectively). OVCAR3 expression was significantly higher than Kuramochi (p = 0.0147; n = 3) [Fig. 2B]. Similarly, OVCAR3 cells exhibited significantly higher FRα gMFI (57125 FU) when compared to Kuramochi (13591 FU; p = 0.0049, n = 3) [Fig. 2B]. TROP-2 expression was highest in SKOV3 cells (99.6% positivity; gMFI 11416 FU), significantly greater than OVCAR3 (64.3%; 1504 FU) and Kuramochi (0.07%; 1869 FU) (p < 0.0001 for all comparisons; n = 3). Kuramochi cells lacked TROP-2 expression [Fig. 2C]. As CLDN6 expression was assessed using an antibody clone recognising an intracellular epitope under non-permeabilising conditions, surface expression was not observed across the three cell lines. Despite this, low-level gMFI signal was observed in Kuramochi (2065 FU) and OVCAR3 (235 FU) cells, which was significantly higher than SKOV3 (12 FU; Kuramochi vs OVCAR3 and Kuramochi vs SKOV3, p < 0.0001; n = 3). OVCAR3 gMFI was also significantly higher than SKOV3 (p = 0.0063; n = 3) [Fig. 2D].

### 3.3 Antigen independent capture and immunofluorescent staining

**Fig. 3:**
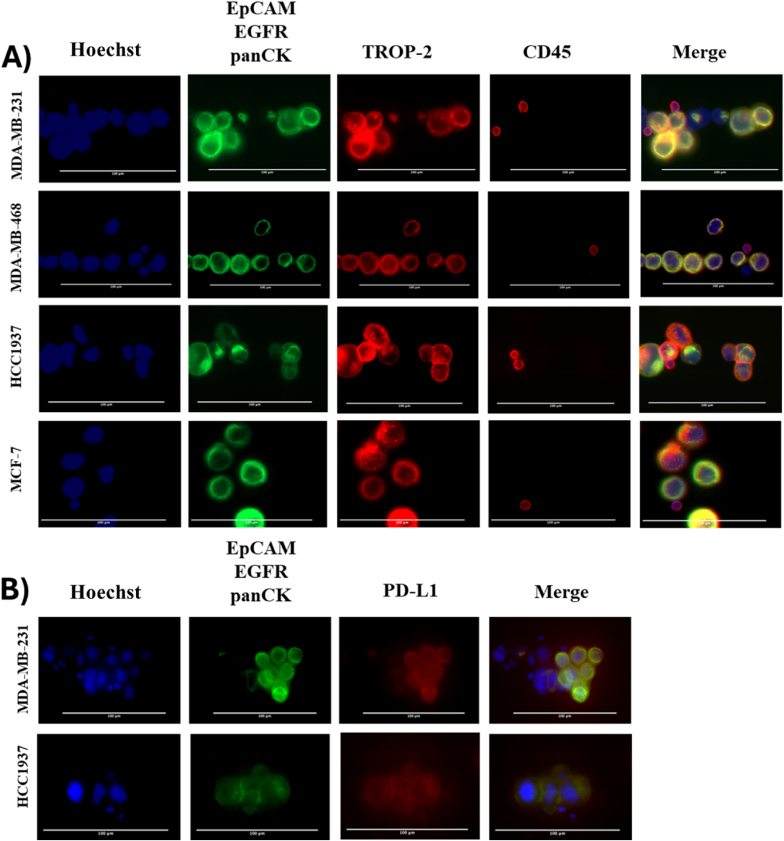
**TNBC cell lines spiked into healthy donor blood and enriched using the Parsortix® system express ADC marker TROP-2 and immunotherapy target PD-L1. A)** TROP-2 expression in TNBC cell lines by immunofluorescence microscopy (n = 3). Cells stained with Hoechst (blue) to visualise nuclei, EpCAM/EGFR/panCK (green) to identify epithelial-origin cells, TROP-2 (red) to assess target antigen expression, and CD45 (far-red) to label and exclude leukocytes. Merged image shows marker co-localisation. Scale bar: 100 μm. **B)** PD-L1 expression in TNBC cell lines by immunofluorescence microscopy. Cell lines were spiked into healthy donor blood and enriched using the Parsortix® system. Cells were stained with Hoechst (blue) to visualise nuclei, EpCAM/EGFR/panCK (green) to identify epithelial-origin cells, PD-L1 (red) to assess target antigen expression, and CD45 (far-red) to label and exclude leukocytes. Merged image shows marker co-localisation. Scale bar: 100 μm.

After confirming target expression by flow cytometry, representative cell lines were used in spike-in experiments to assess detection with the optimised CTC enrichment and staining workflow. Cultured cells were spiked into healthy donor blood and processed using the Parsortix® system, followed by in-cassette staining to optimise staining conditions prior to patient sample analysis. Enriched cells were stained for nucleus (Hoechst, blue), epithelial markers EpCAM/EGFR/panCK (green), TROP-2 (red), and leukocyte marker CD45 (far-red). As demonstrated in Fig. 3A, all three TNBC cell lines demonstrated positive staining for epithelial markers (EpCAM, EGFR and panCK) and TROP-2. A small CD45+ population was detected, and the absence of overlap between positive and negative selection markers indicates distinct populations. As shown in Fig. 3B, spiked MDA-MB-231 were enriched and stained for PD-L1 (red) together with EpCAM/EGFR/panCK (green) and Hoechst (blue). Both cell lines were positive for PD-L1 (red) and the epithelial markers (green). PD-L1 expression was observed in both cell lines, consistent with flow cytometry.

**Fig. 4:**
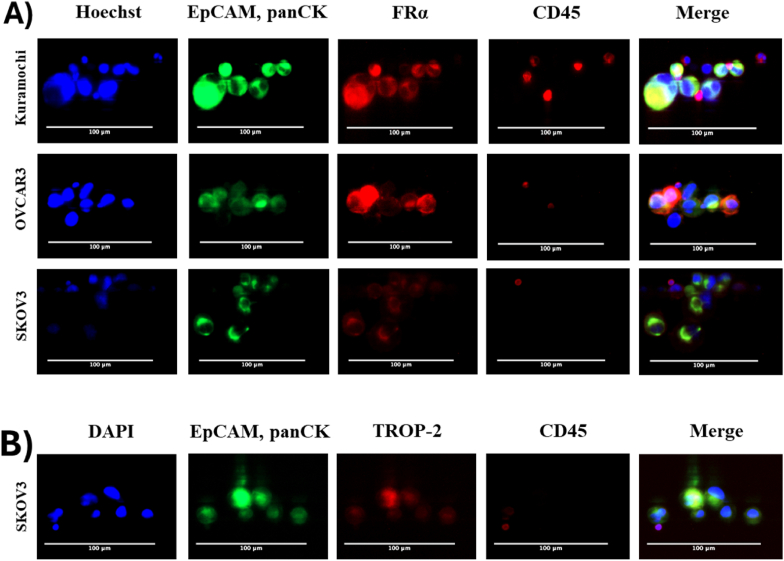
**EOC cell lines spiked into healthy donor blood and enriched using the Parsortix® system express the ADC target FRα. A)** Cells stained with Hoechst (blue) to visualise nuclei, EpCAM/panCK (green) to identify epithelial-origin cells, FRα (red) to assess target antigen expression, and CD45 (far-red) to label and exclude leukocytes. Merged image shows marker co-localisation. Scale bar: 100 μm. **B)** SKOV3 cell line spiked into healthy donor blood and enriched using the Parsortix® system. Cells stained with Hoechst (blue) to visualise nuclei, EpCAM/panCK (green) to identify epithelial-origin cells, TROP-2 (red) to assess target antigen expression, and CD45 (far-red) to label and exclude leukocytes. Merged image shows marker co-localisation. Scale bar: 100 μm.

Representative EOC cell lines Kuramochi, OVCAR3 and SKOV3 were used in spike-in experiments to evaluate detection within the optimised CTC enrichment and staining workflow. Cultured cells were spiked into healthy donor blood and processed through the Parsortix® system, followed by in-cassette fixation and immunofluorescent staining, as described. This enabled optimisation of staining conditions prior to patient sample analysis. As shown in Fig. 4, all three EOC cell lines demonstrated positive staining for epithelial markers (EpCAM and panCK), with the Kuramochi cells showing the strongest epithelial signal intensity, followed by OVCAR3 and SKOV3. CD45+ leukocytes were distinctly identified in all preparations, confirming specificity and allowing clear discrimination between tumour and immune cell populations. All three cell lines exhibited detectable FRα expression (Fig. 4A), however, Kuramochi and OVCAR3 cells displayed markedly higher fluorescence intensity compared to SKOV3. SKOV3 cells were further assessed for TROP-2 expression, as they were identified as the cell line with the highest expression by flow cytometry. Immunofluorescent staining confirmed TROP-2 signal on SKOV3 cells (Fig. 4B).

**Fig. 5:**
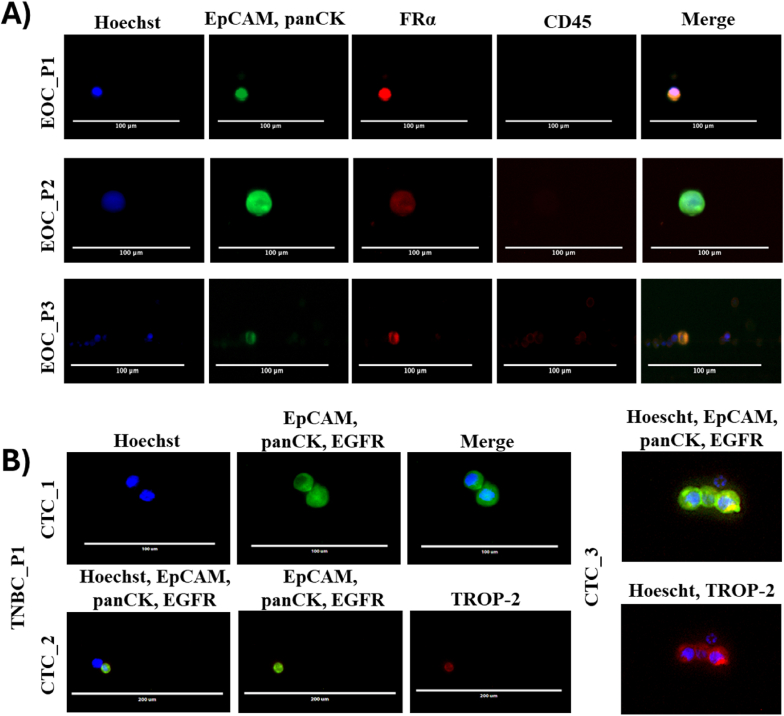
**Parsortix®-enriched EOC and TNBC CTCs. A): EOC cancer patients**: CTCs enriched from whole blood using Parsortix®, fixed and stained in-cassette with Hoechst (blue; nuclei), EpCAM/pan-cytokeratin (panCK; green; epithelial identity), FRα (red; target antigen), and CD45 (far-red; leukocytes for exclusion). Merged images show marker co-localisation on CTCs; CD45+ cells are leukocytes. Rows: Patient 1 (top), Patient 2 (middle), Patient 3 (bottom). Scale bar: 100 μm. **(B) TNBC patient:** Parsortix®-enriched CTCs stained with EpCAM/EGFR/panCK/CK19 (green) and TROP-2 (red); Hoechst (blue) marks nuclei and CD45 (far-red) labels leukocytes. Panels are organised by individual CTCs as follows: CTC_1 (top row; left to right): Nuclear stain (Hoechst, blue); epithelial stain (EpCAM/EGFR/panCK/CK19, green); merge (Hoechst + epithelial). CTC_2 (middle row; left to right): Composite (Hoechst + epithelial); epithelial stain (green); TROP-2 stain (red). CTC_3 (Right side; stacked): Top-composite (Hoechst + epithelial); bottom-composite (Hoescht + TROP-2 stain) (red).

Following optimisation of enrichment and staining conditions through cell line spike-in experiments, CTCs were analysed from a pilot cohort of patients with advanced EOC (n = 3, stage III) and metastatic TNBC (n = 6). In EOC patients, CTCs were successfully enriched from peripheral blood and identified as Hoechst+/EpCAM+/panCK+/CD45-cells. FRα expression was detectable on CTCs from all three EOC patients (Fig. 5A). In TNBC, TROP-2+ CTCs were identified in patient samples (Fig. 5B). These findings demonstrate the feasibility of detecting clinically relevant ADC target expression on patient-derived CTCs using this label-independent enrichment.

## 4 Discussion

This study demonstrates substantial heterogeneity in the expression of clinically actionable ADC targets across TNBC and EOC models and establishes a practical workflow for assessing these targets on CTCs. A central challenge in ADC deployment is that single, often archival, tissue biopsies rarely capture spatiotemporal heterogeneity or on-treatment evolution, risking suboptimal patient selection and limited insight into resistance. By contrast, CTCs enable minimally invasive assessment of target availability on viable tumour cells, offering a window into evolving drug-target biology. A key strength of this workflow is the use of the Parsortix® system, which enriches CTCs based on their biophysical properties (size and deformability) rather than surface markers, and has been previously applied to assess HER2 expression on mBC CTCs [26]. Label-independent enrichment is particularly relevant given our observation that EpCAM expression varied substantially across the cell lines tested. In the mesenchymal-like TNBC line MDA-MB-231, EpCAM positivity was markedly reduced (31%) compared to other lines. The HGSOC model Kuramochi also demonstrated low EpCAM expression (35%). We have previously demonstrated the prognostic significance of Parsortix®-enriched CTCs in advanced HGSOC, and the present work extends this platform to ADC target assessment [23].

TROP-2 was uniformly expressed across all TNBC cell lines, including the EpCAM-low MDA-MB-231, consistent with recent reports demonstrating sustained TROP-2 expression on CTCs even when EpCAM is downregulated [31]. The preservation of TROP-2 in EpCAM-low cell lines supports a potential dual role for TROP-2, both as a therapeutic ADC target and as a supplementary marker for CTC detection in phenotypically diverse tumour populations. However, it should be noted that formal EMT characterisation was not performed in this study, and the classification of MDA-MB-231 as mesenchymal-like is based on its established molecular features rather than direct assessment of EMT markers on the captured cells.

The flow cytometry data revealed substantial heterogeneity in ADC target expression across the cell line panels. In TNBC, FRα was largely absent, consistent with the low FRα positivity rates (∼10%) in 96 screened TNBC patients (≥25% of cells having ≥1+ membranous expression by IHC) screened in a phase II study of mirvetuximab soravtansine [35]. In EOC models, FRα and TROP-2 expression profiles differed across subtypes. SKOV3 and OVCAR3 demonstrated moderate FRα positivity and detectable TROP-2, whereas the HGSOC line Kuramochi lacked TROP-2 expression and showed reduced FRα positivity, suggesting that target availability may be subtype-dependent. Such cell line-level heterogeneity is in line with clinical observations that antigen expression in TNBC and EOC can vary between patients and evolve under treatment pressure [9, 12]. These observations support a rationale for expanding this workflow towards serial monitoring of ADC target status. By immunofluorescence, Kuramochi and OVCAR3 cells displayed markedly higher signal intensity than SKOV3, potentially signifying a difference in intracellular vs extracellular folate distribution.

PD-L1 expression was heterogeneous across the TNBC cell lines, with high positivity in MDA-MB-231 and HCC1937 and low expression in MDA-MB-468. These patterns are broadly consistent with known subtype-linked immune phenotypes in TNBC [36]. The co-detection of PD-L1 alongside TROP-2 on enriched cells is notable given recent clinical evidence that ADC–immunotherapy combinations can improve outcomes. The ASCENT-04/KEYNOTE-D19 trial demonstrated that sacituzumab govitecan combined with pembrolizumab improved outcomes compared to chemotherapy plus pembrolizumab in previously untreated PD-L1+ advanced TNBC [19]. Immune checkpoint inhibitors are now being incorporated into first-line neoadjuvant treatment for TNBC [32], and the ability to co-assess ADC targets and immune checkpoint markers on CTCs could support patient stratification for combination regimens. However, our observations are based on cell line models, and further clinical validation is required to determine whether CTC-based PD-L1 and TROP-2 co-expression can predict benefit from combination approaches. A practical constraint of four-colour fluorescent microscopy is that channels allocated to nuclear, epithelial, and immune cell markers leave only a single experimental channel, limiting co-expression analysis.

CLDN6 remains a target of active clinical interest, with CLDN6-directed ADCs (TORL-1-23, DS-9606a) and cellular therapies (BNT211-01) currently in early-phase trials [33,34]. Given the use of an antibody clone recognising an intracellular epitope under non-permeabilising conditions in this study, the low gMFI patterns observed may reflect residual intracellular staining rather than true surface expression. Ongoing work aims to optimise antibody clones directed against extracellular epitopes that align with the therapeutic ADC binding site, enabling reliable incorporation of CLDN6 into CTC-based workflows.

Recent evidence from mBC suggests that epitope downregulation is not a predominant mechanism of ADC resistance [37]. In a study monitoring CTCs from 35 patients receiving ADC therapies, both TROP-2 and HER2 expression generally persisted or even increased at disease progression, suggesting that treatment failure was more often associated with resistance to the payloads rather than loss of the targeted epitopes [37]. CTC-based monitoring could therefore help distinguish between these resistance mechanisms by enabling longitudinal, paired assessment of CTC numbers and target antigen expression, disease progression with preserved target expression would suggest payload or intracellular resistance, whereas progression with reduced expression would favour antigen-driven escape.

The clinical precedent for CTC-guided targeted therapy is growing. In the DETECT III trial, patients with HER2-mBC but HER2+ CTCs showed improved overall survival when treated with lapatinib [27]. Similarly, the CirCe T-DM1 trial demonstrated that HER2-amplified CTCs could be used to identify candidates for trastuzumab emtansine therapy among patients with HER2-tumours, although objective responses were limited [38]. These studies illustrate how CTC molecular profiles can extend treatment eligibility beyond what tissue-based assessments would indicate and support the incorporation of CTC-based ADC target analysis into prospective clinical trial designs. As ADCs emerge as a therapeutic option, there will be an increasing need to standardise liquid biopsy approaches to ensure adherence to international standards and enable their reliable clinical translation [39].

This study has several limitations. The patient cohort was small (n = 6 TNBC, n = 3 EOC), reflecting the proof-of-concept nature of this workflow establishment study. The cross-sectional design with single-timepoint sampling limits our ability to assess temporal dynamics of target expression, and we did not correlate CTC target expression with matched tissue-based IHC results or clinical outcomes. Future studies with larger cohorts, longitudinal sampling during ADC therapy, and direct tissue–CTC comparisons are warranted to establish the predictive and prognostic value of CTC-based ADC target monitoring.

## 5 Conclusion

This study demonstrates the technical feasibility of assessing ADC targets on CTCs using label-independent enrichment and multiplex immunofluorescence in TNBC and EOC. The workflow enables simultaneous assessment of multiple clinically relevant targets, including TROP-2 and FRα, as well as the immune checkpoint marker PD-L1, on viable tumour cells obtained through minimally invasive blood sampling. As the ADC therapeutic landscape continues to expand, prospective integration of CTC-based target monitoring into clinical trial designs, with predefined timepoints and response-correlation endpoints, will be essential to validate this approach for treatment-guiding clinical practice.

## Declaration of competing interest

The authors declare the following financial interests/personal relationships which may be considered as potential competing interests: Karen Cadoo reports a relationship with MSD Ireland Ballydine that includes: funding grants and speaking and lecture fees. Karen Cadoo reports a relationship with ImmunoGen Inc that includes: funding grants. Karen Cadoo reports a relationship with Karyopharm Therapeutics Inc that includes: funding grants. Karen Cadoo reports a relationship with GSK Ireland that includes: consulting or advisory. Karen Cadoo reports a relationship with Verastem Inc that includes: funding grants. Karen Cadoo reports a relationship with NextCure Inc that includes: consulting or advisory. Karen Cadoo reports a relationship with Ipsos that includes: consulting or advisory. Karen Cadoo reports a relationship with Fresh Perspectives that includes: consulting or advisory. Karen Cadoo reports a relationship with MJH Life Sciences LLC that includes: speaking and lecture fees. Karen Cadoo reports a relationship with Astra Zeneca that includes: speaking and lecture fees. Karen Cadoo reports a relationship with Roche Ireland Ltd that includes: travel reimbursement. Karen Cadoo reports a relationship with Eisai Inc that includes: non-financial support. Karen Cadoo reports a relationship with Arc Cancer Support Centers that includes: board membership. Karen Cadoo reports a relationship with NRG Oncology Foundation Inc that includes: funding grants. If there are other authors, they declare that they have no known competing financial interests or personal relationships that could have appeared to influence the work reported in this paper.
